# An Editor-in-Chief’s farewell

**DOI:** 10.1007/s00424-024-03054-4

**Published:** 2024-12-12

**Authors:** Armin Kurtz

**Affiliations:** Institute of Physiology, University of Regensburg, Regensburg, Germany

It was a great honor and also a pleasure for me to serve *Pflügers Archiv—European Journal of Physiology* (PA-EJP) as an Editor-in-Chief for 9 years. *Pflügers Archiv* was founded by the German physiologist Eduard Pflüger. It is the world’s oldest and still active publication journal in the field of physiology. I had the pleasure to celebrate the 150th anniversary of the journal during my term as EiC [2]. Even in times of rapidly changing publication scenarios, *Pflügers Archiv*-European Journal of Physiology remained as a stable rock in the surf for authors. Without disregarding or neglecting the psychological impact of the journal impact factor on scientists in the fields of physiology or medicine, it was the journal’s primary strategic aim to provide an honest and trustful publication platform for research in the field of (translational) physiology. The main criteria for published papers were originality and reproducibility of results. Estimation of these parameters becomes increasingly difficult with the developing potential misuse of artificial intelligence. Therefore, the work of our expert reviewers cannot be appreciated enough in this context. My sincere thanks therefore go to the editorial board members of our journal and to external reviewers for their excellent and important work for the journal. In line with this, I further wish to express my deep gratitude to our Associate Editors who essentially contributed to the performance of our journal by thorough handling of manuscripts and contributing attractive reviews and Special Issues. We had a great, reliable, and trustful collaboration within the Board of Associate Editors which was an essential element for my work as EiC. Finally, I wish to thank the Journal Editors from Springer publisher for the very friendly and constructive collaboration which we had.

A real highlight of my work as EiC was the establishment of a special partnership between PA-EJP and the German Physiological Society (DPG) [1]. PA-EJP has become the home-journal of the DPG which means a win–win situation for both partners. This union allows a promising look into the future. With this positive aspect in mind, I am delighted to hand over the Editor-in-Chief position to my successor CARSTEN WAGNER with my best wishes for further success.

## References

[1] Hecker M, Kurtz A (2020) Pflügers Archiv-European Journal of Physiology becomes the official journal of the German Physiological Society. Pflugers Arch - Eur J Physiol 472:1657. 10.1007/s00424-020-02493-z

[2] Kurtz A (2018) Celebrating 150-year anniversary! Pflugers Arch-Eur J Ühysiol 470(12):1719–1720. 10.1007/s00424-018-2220-2 30324320 10.1007/s00424-018-2220-2

